# Natural killer cell-derived exosome-based cancer therapy: from biological roles to clinical significance and implications

**DOI:** 10.1186/s12943-024-02045-4

**Published:** 2024-06-29

**Authors:** Chaohua Si, Jianen Gao, Xu Ma

**Affiliations:** National Research Institute for Family Planning, Chinese Academy of Medical Sciences & Peking Union Medical College, Beijing, 100000 China

**Keywords:** Natural killer cells, Cancer therapy, Natural killer cell-derived exosomes, Modification, Clinical translation

## Abstract

Natural killer (NK) cells are important immune cells in the organism and are the third major type of lymphocytes besides T cells and B cells, which play an important function in cancer therapy. In addition to retaining the tumor cell killing function of natural killer cells, natural killer cell-derived exosomes cells also have the characteristics of high safety, wide source, easy to preserve and transport. At the same time, natural killer cell-derived exosomes are easy to modify, and the engineered exosomes can be used in combination with a variety of current cancer therapies, which not only enhances the therapeutic efficacy, but also significantly reduces the side effects. Therefore, this review summarizes the source, isolation and modification strategies of natural killer cell-derived exosomes and the combined application of natural killer cell-derived engineered exosomes with other antitumor therapies, which is expected to accelerate the clinical translation process of natural killer cell-derived engineered exosomes in cancer therapy.

## Introduction

More than four decades ago, natural killer (NK) cells were identified as lymphocytes with the innate ability to lyse tumor cells, destroying target cells without prior sensitization, without MHC restriction, and with a fast response rate, acting early in the immune response, and being responsible for killing abnormal cells such as aging cells and tumor cells in the body [1–4].Natural killer cells are a specific immune effector cell type which is an important component of tumor immune surveillance, plays a key role in immune activation against aberrant cells, has innate virulence and immunomodulatory capacity, and accounts for 5–20% of circulating lymphocytes in the body [5].

With a deeper understanding of natural killer cells, researchers have come to realize that natural killer cell-derived exosomes have many unique advantages in addition to retaining most of their tumor-killing functions [6, 7]. For example, it is easy to modify, including physical modification, chemical modification, biological modification and immune modification, to enhance certain properties of natural killer cell-derived exosome [8, 9]. Second, Natural killer cell-derived exosomes are relatively safe in tumor therapy, as cell-based therapies, including NK cell-based therapies, carry the risk of triggering a “cytokine storm,” which may force patients to suspend treatment and, in some cases, may even be life-threatening; however, the use of natural killer cell-derived exosomes may not be accompanied by such serious side effects [10, 11]. Meanwhile, compared with natural killer cells, natural killer cell-derived exosomes are also widely available and easy to store and transport [12, 13]. These advantages make natural killer cell-derived exosomes show a strong potential for clinical application.

What is more, natural killer cell-derived exosomes can be used as a new drug delivery platform, which, in addition to their own anti-tumor efficacy, can also reduce the side effects of treatments such as radiotherapy [14]. Meanwhile, in the process of tumor treatment, we have already realized that each tumor treatment method has its own advantages and disadvantages, and a “one-size-fits-all” treatment method does not yet exist [15]. Therefore, researchers must choose to integrate multiple strategies to enhance the effectiveness of tumor therapy, and natural killer cell-derived exosomes have the ability to work in conjunction with other tumor treatments, which are often more effective and have fewer side effects [16]. In conclusion, this paper reviews the roles of natural killer cell-derived exosomes, working modes, and recent advances in engineered exosomes, as well as summarizes the clinical value and application of natural killer cell-derived exosomes based on natural killer cell-derived exosomes, which is expected to accelerate the clinical translation of natural killer cell-derived exosomes in targeted cancer therapies.

## Origin of natural killer cell and cancer immunotherapy

Natural killer cells come from a wide range of sources and can currently be obtained from peripheral blood, cord blood, pluripotent stem cells and NK cell lines (Table 1) [17]. Based on their sources and efficacy in cancer treatment, several NK cell lines have been established internationally over the years, including HANK1, KHYG-1, NK92, NK92MI, NKL, NKT, NK-YS and YT, these cell lines have often contain different molecules that are capable and therapeutic potential, greatly expanding the scope of action of natural killer cells [18]. This review will summarize the origins as well as the role of natural killer cells in cancer immunotherapy.

Natural killer cells originate from common lymphoid progenitors (CLP), pass through a common ILC precursor stage (CILCP), and eventually develop into NK progenitors (NKP) [19]. Natural killer cells are characterized by the expression of CD122, loss of CD34 and CD127, and its further differentiation is dependent on the expression of T-bet and Eomes. Based on CD56 expression, human NK cells are usually subdivided into two main subpopulations: the CD56^bright^CD16^dim/−^ and the CD56^dim^CD16, the former traditionally considered to be more cytokine-producing and less mature, and the latter, more cytotoxic and maturer Higher. Both subpopulations express the activating surface receptors NKp4 and NKp80, and the transformation of CD56^bright^ NK cells to CD56^dim^ NK cells is achieved by expression of CD16, PEN5, and CD57 [5, 19–21]. The bone marrow is the primary site of natural killer cell development and may also develop and mature in stimulated lymphoid organs, including the tonsils, spleen, and lymph nodes [22]. Subsequently, based on their tissue-resident properties, natural killer cells can be classified into classical natural killer cells with cycling properties and tissue-resident natural killer cells [23].

Only activated natural killer cells can function [24]. The surface of natural killer cells contains a range of inhibitory and activating surface receptors, and its activation is dependent on ligand/receptor interactions, the dynamic balance between inhibitory and activating receptors on the cell surface ensures the regulation of NK cell effector functions (Table 2) [25]. Healthy cells express few ligands that activate natural killer cells, but express high levels of major histocompatibility complex class I molecules (MHC I), which bind to the killer immunoglobulin-like (KIR) family of inhibitory receptors on natural killer cells in order to protect them from natural killer cell attack [26]. In tumor cells, the expression level of MHC I is down-regulated, and the expression level of natural killer cell-activating ligands is up-regulated, thereby activating natural killer cells [27].

**Fig. 1:**
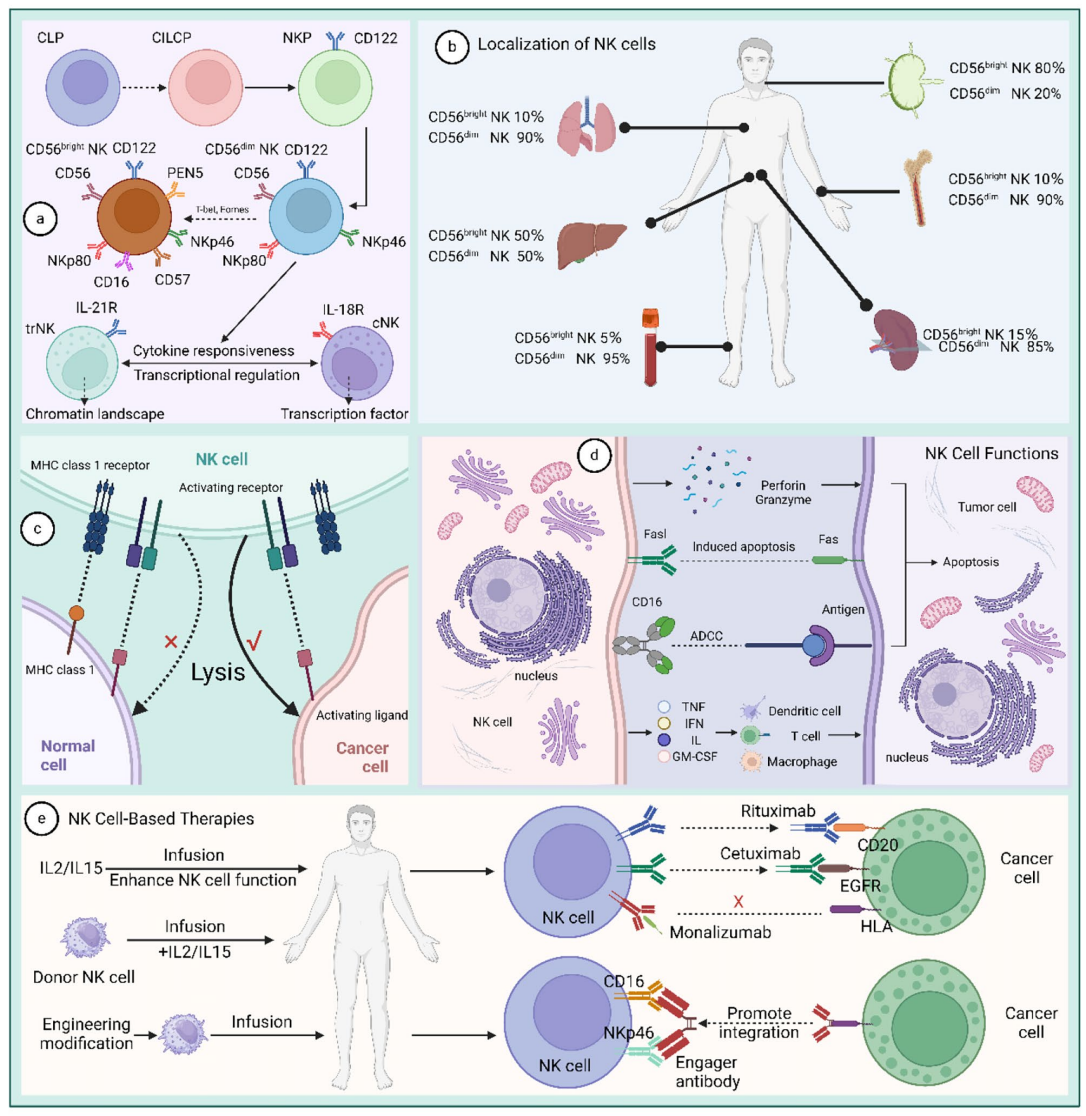
Origin of natural killer cell and cancer immunotherapy. (**a**) The origin of natural killer cells. (**b**) Localization of NK cells. (**c**) Natural killer cells function selectively. (**d**) Four ways natural killer cells fight tumors. (**e**) Natural killer cell-based therapies

Due to these properties, natural killer cell-based immunotherapies are gradually being developed. The first NK cell-based therapies were discovered in hematopoietic stem cell transplants (HSCTs), in which NK cells have the ability to exert graft-versus-leukemia effects. A study by Ruggeri et al. found that KIR-mismatched allogeneic-reactive donor NK cells protected bone marrow transplanted AML patients from AML relapse while avoiding graft-versus-host disease (GVHD) [28, 29]. The study by Miller et al. pioneered the use of natural killer cells in a non-transplant setting, further demonstrating the impact of effective lymphocyte depletion preconditioning on NK cell expansion and persistence in vivo [30]. This was followed by the gradual emergence of Chimeric Antigen Receptor (CAR)-NK cells as an alternative to CAR-T therapy and the mainstream use of natural killer cells for immunotherapy [5]. With new tools for genetic engineering approaches and new understanding of NK cell biology, NK-based immunotherapies are bound to show greater potential in preclinical and clinical development. Also, natural killer cell-derived exosomes will be used for cancer immunotherapy (Fig. 1).

**Table 1:** Comparison of commonly used allogeneic NK cell sources

NK cell source	Advantages	Limitations	Status of CAR NK cell immunotherapy programme
Cord blood	Easily collected and highly amplified in vivo	Heterogeneous with poor genetic modification	Reported [31]
Pluripotent stem cells	High proliferation capacity, homogeneous cell population, ability to cryopreserve UCBs	Immature phenotype, only modified to fulfill ADCC role	Reported [32]
Peripheral blood	Mature phenotype, highly cytotoxic	Requires in vivo amplification and is not readily obtained	Unreported
NK cell line	Homogeneous cell population, can give multiple doses, easy to expand, easy to genetically modify	Lack of certain receptors, no ADCC effect, limited number of generations amplified in vivo	Unreported

**Table 2:** Common human NK cell receptors and their ligands

Active/ Inhibitory	Receptor	Ligands
Active	NCR1	HS GAGs, Complement factor P, vimentin, viral HA
	NCR2	PCNA, Syndecan-4, Nidogen-1, viral HA, HS GAGs, PDGF-DD, 21spe-MLL5
	NCR3	B7-H6, galectin-3, GAGs, viral hemagglutinin, pp65
	CD16	Fc portion of IgG antibodies
	NKG2D	MICA, MICB and UL16-binding proteins
	DNAM1	PVR, nectin-2
Inhibitory	KIR2DL1	HLA-C, group 2
	KIR2DL2/3	HLA-C, group 1
	KIR3DL1	HLA-Bw4
	KIR3DL2	HLA-A3, A11
	NKG2A	HLA-E

## Spatio-temporal specificity of natural killer cell-derived engineered exosomes

### Isolation of natural killer cell-derived exosomes

The isolation of exosomes is important for the study of mechanistic and clinical research. Currently, popular methods of exosome isolation include ultracentrifugation, kit extraction, ultrafiltration, precipitation, and so on. Among them, the immunosorbent method utilizes antigen-antibody interactions to extract exosomes, which is not only highly specific, but also yields a higher rate of exosomes than ultracentrifugation [33]. To further improve this method, Li et al. utilized sub-micron level magnetic particles for immunoaffinity, which resulted in exosome yields that were 10–15 times higher than the ultracentrifugation method [34]. The immunoaffinity method has no body level limitation and has potential for clinical application, but the high cost and complexity of the technique limit its use in the clinic [33].

Recently, a microfluidic technique based on fluid properties for exosome separation has gradually come into the limelight. Microfluidics enables a fusion of traditional and emerging technologies, including the usual separation determinants such as size, density, and immunoaffinity, as well as innovative sorting mechanisms such as electromagnetic manipulation, nanowire-based traps, acoustics, electrophoresis, nano-size-deterministic transverse displacement, and viscoelastic flow [35, 36]. For example, the micro- and nanofluidic device designed by Davies RT et al. uses mis-flow filtration to separate and capture liposarcoma-derived exosomes. Its combination of differently sized separation units and CD63-based antibody immunoaffinity resulted in a 5-fold increase in the number of key liposarcoma-associated extracellular vesicle cargoes within 30 min [36–38].

**Fig. 2:**
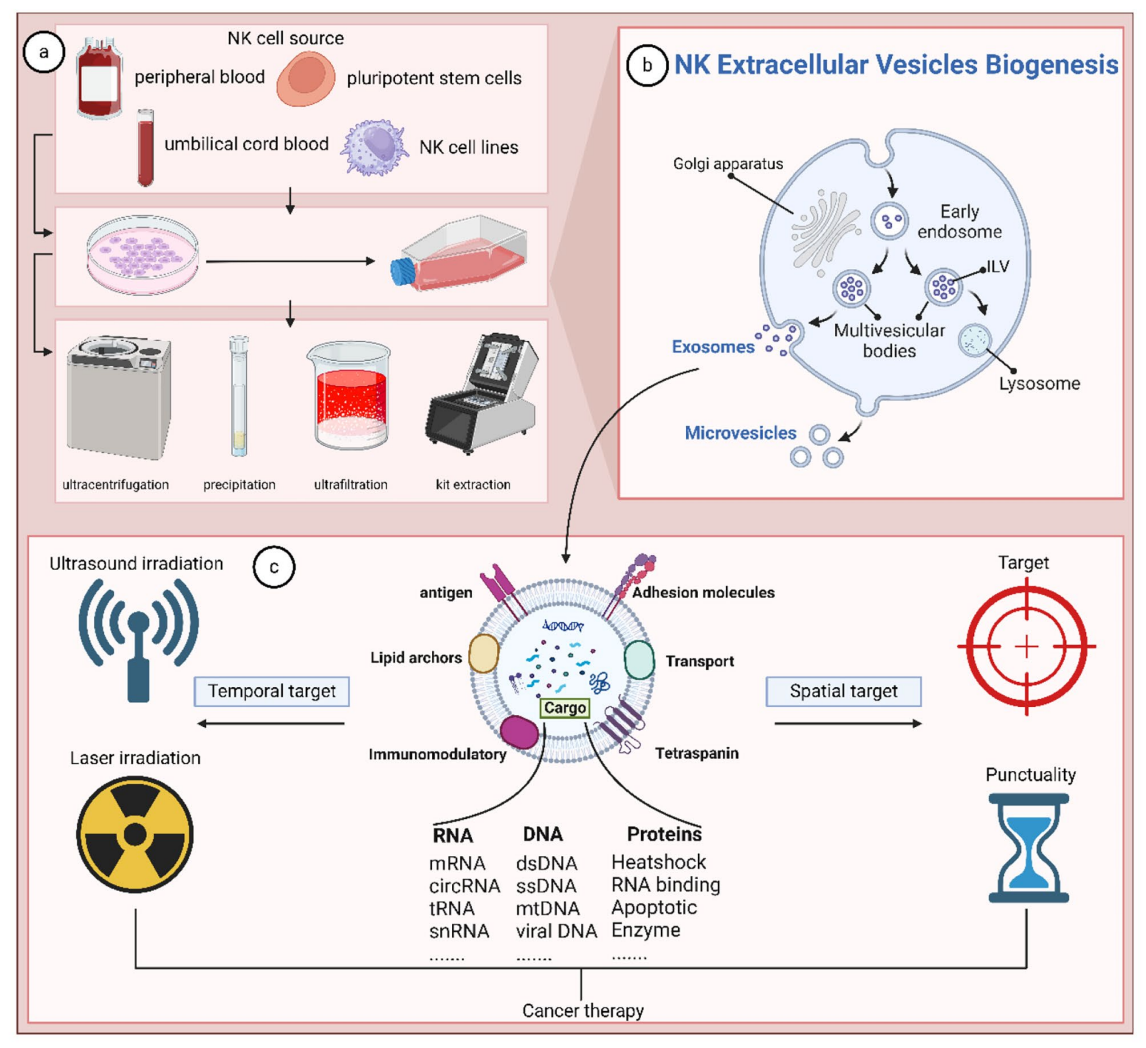
Spatio-temporal specificity of natural killer cell-derived engineered exosomes. **(a)** Sources and methods of isolation of natural killer cell-derived exosomes. **(b)** Biogenesis of natural killer cell-derived exosomes. **(c)** Natural killer cell-derived exosomes are spatiotemporally specific in their action

There is no absolute best isolation method for exosomes, and its isolation results are closely related to downstream applications and scientific issues, but high recovery and high specificity are two recognized basic requirements [39]. At the same time, we also have to consider purity, yield, cost and many other aspects. Therefore, conventional methods alone usually cannot meet the separation requirements, and the integration of basic methods such as microfluidics is essential for the separation of exosomes with high purity and high yield (Table 3) (Fig. 2).

**Table 3:** Common Exosome Isolation Techniques

Technique	Specificity/ Recovery rate	Advantages	Limitations	Reference
Asymmetric flow field-flow fractionation	High/Low	Time-saving, reproducible, fully automated assay, simulates physiological conditions	Small sample volume and low throughput	[40, 41]
Density gradient centrifugation	High/Low	Higher purity and biostructural integrity	Preliminary work is tedious, complicated, time-consuming, and difficult to remove high-density chemicals.	[42–44]
Hydrostatic filtration dialysis	Middle/ Middle	Low cost, no chemical contamination, high throughput	Extraction requires additional sterilization and is less efficient with larger sample sizes	[45]
Immunoaffinity-based isolation methods	High/Low	High purity, high specificity, good integrity of isolated exosomes	High cost, low throughput and separation efficiency, only for cell-free samples	[46]
Mass density-based ultracentrifugation	Low/ High	Simple operation, high throughput, no chemical contamination	Instruments are costly, time-consuming, and prone to mixing with other types of EVs to interfere with subsequent analyses.	[47–50]
Magneto-immunoprecipitation	High/Low	Simple operation and good reproducibility	High cost, low flux, low biological activity	[34]
Microfluidic	High/Low	Portability and purity with low reagent and sample consumption to combine exosome extraction and analysis	Lack of standardized methodology, high cost, complex equipment, low throughput	[51, 52]
Size-based isolation methods Ultrafiltration	Middle/ Middle	Low instrument requirements, no chemical reagent pollution, time-saving and efficient	Soluble proteins are difficult to remove, the purity, shape and charge of the sample can affect the separation, low biological activity	[53]
Size-exclusion chromatography	Middle/ Middle	High purity, sensitivity, integrity and bioactivity without chemical contamination	High instrument cost, low throughput, time-consuming, low purification yields	[54, 55]
Precipitation	Low/ High	High yield, high recovery, high integrity without special instruments or techniques	Complicated sample preparation procedures, difficult to remove isolated exosomal lipoproteins, non-uniform particle size, exosomes are easily damaged	[33, 56]

### Spatio-temporal specificity

Exosomes of natural killer cell origin containing specific targeting molecules modified to enable precise drug delivery. At the same time, the point in time at which the engineered exosomes release their cargo can be controlled by additional physical influences, such as laser and ultrasound radiation [16]. Whiteside et al. demonstrated that exosomes of all cell origins can cross the blood-brain barrier at different rates [57]. Liu et al. designed Exo^CAR/T7^@Micelle (where Micelle is PEG-TK-Ce6@RSL3) that allows them to precisely target to HER2-positive breast cancer cells. Compared to exosomes without targeting molecules, engineered exosomes trigger more intense tumor. Meanwhile, for internal micellar structures, the Photodynamic therapy (PDT) strategy was used to generate cytotoxic reactive oxygen species (ROS) and facilitate ROS-triggered drug release with the help of the photosensitizer chlorine e6 (Ce6) and the ROS-sensitive linkage of the chemical thioketone of bone (TK) [16]. The above engineering modifications enabled the treatment of HER2-positive breast cancer brain metastases.

Taken together, the targeted delivery of engineered exosomes of natural killer cell origin depends on surface targeting molecules that allow full access of the exosomes into tumor cells and lower concentrations in healthy organs. And by additional physical influences the engineered exosomes release biologically active therapeutic molecules at specific times. Thus, engineered exosomes of natural killer cell origin are able to prolong the survival time of tumor patients and are expected to open new frontiers for modern drug delivery (Fig. 2).

## Natural killer cell-derived engineered exosomes can serve as drug delivery platforms

### Natural killer cell-derived exosomes exert tumor-killing functions

Natural killer cell-derived exosomes are able to kill tumors through several pathways. One, natural killer cell-derived exosomes contain a variety of cleavage particles (perforin, granzyme, granulocyte fusion) that induce target cell death [58]. Second, natural killer cell-derived exosomes contain a series of cytokines (TNF-α, IL-10, IFN-Y), chemokines (CCL3, CCL4, CCL5, XCL1), and growth factors (GM-CSF), which can interact with macrophages and dendritic cells to exert immune-response effects [59]. Third, natural killer cell-derived exosomes contain multiple members of the tumor necrosis factor superfamily, such as FASL, which can induce apoptosis in target cells by binding to the corresponding receptor (such as FAS) [60]. Fourth, natural killer cell-derived exosomes can also mediate antibody-dependent cell-mediated cytotoxicity (ADCC), which mediates the direct action of killer cells on target cells. Fifth, natural killer cell-derived exosomes contain a variety of therapeutic molecules such as miR-1249-3p [61]. Thus, natural killer cell-derived exosomes can themselves be used as a drug for tumor therapy.

### Strategies for engineering modification of natural killer cell-derived exosomes

**Fig. 3:**
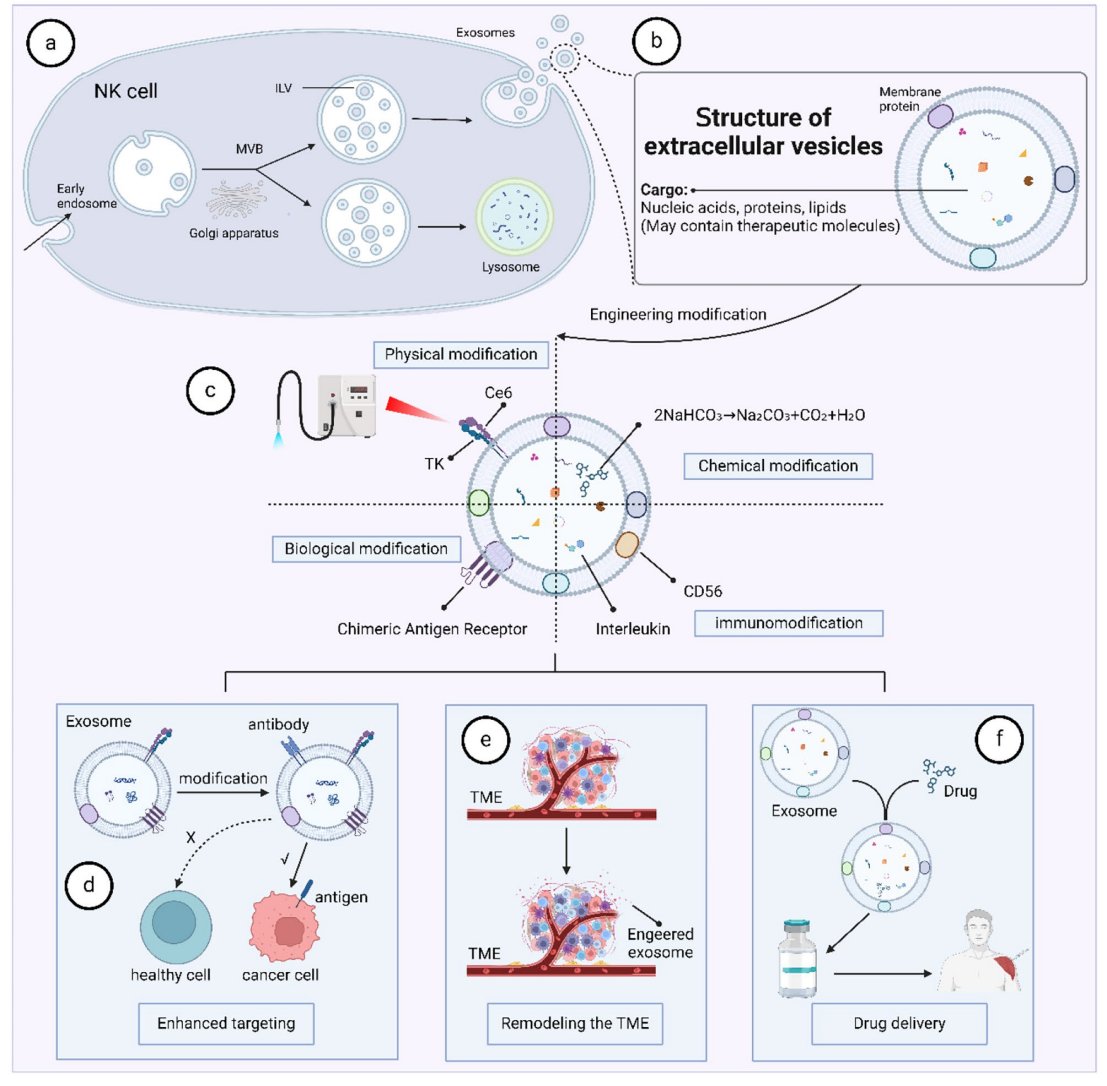
Natural killer cell-derived engineered exosomes can serve as drug delivery platforms. (**a, b**) Source and structure of natural killer cell-derived exosomes. (**c**) Engineering modification strategies for natural killer cell-derived exosomes. (**d, e, f**) Engineering modification strategies to confer or enhance novel properties of natural killer cell-derived exosomes

A variety of modifications can be added to natural killer cell-derived exosomes to enhance or confer multiple capabilities. For one, natural killer cell-derived exosomes containing physical modifications enable more precise tumor-targeting effects, such as laser irradiation. Liu et al. fused natural killer cell-derived exosomes with the photosensitizer chlorine e6 (Ce6) and ROS-sensitively linked chemical thioketone (TK), and in the case of laser irradiation the TK was destroyed by the ROS generated by Ce6. As a result, exosomes containing therapeutic molecules can be delivered and released with precision [16]. Second, exosomes can be chemically modified. For example, Thuy et al. encapsulated NaHCO3 (sodium bicarbonate, SBC) and paclitaxel into exosomes, which produce carbon monoxide and cleave when swallowed by tumor cells, thereby effectively releasing the drug [62]. Third, natural killer cell-derived exosomes can be biologically modified, and the most widely used strategy is membrane modification to enhance targeting. Liu et al. fused natural killer cell-derived exosome membranes with CAR modification and a T7 peptide (sequence HAIYPRH) to achieve precision delivery of engineered exosomes [16]. Fourth, natural killer cell-derived exosomes can be immunomodified to enhance their anti-tumor immune function.CD56 is not only a marker of natural killer cells, but also has powerful immunostimulatory effector functions, including the production of paracrine T-cell factor 1 and highly efficient cytotoxicity [63]. Fabbri et al. used CD56 antibody to modify natural killer cell-derived exosomes and enhanced the natural cytotoxicity of natural killer cell-derived exosomes and ultimately treated neuroblastoma by combination therapy [64]. In conclusion, natural killer cell-derived exosomes have the advantages of easy operation, high safety, and wide source, etc. In addition to their own tumor-killing function, they are also very suitable to be used as drug delivery carriers, which is expected to open up a new platform for future drug delivery (Fig. 3).

## Synergies of engineered natural killer cell-derived exosomes with other tumor therapies

### Radiotherapy

**Fig. 4:**
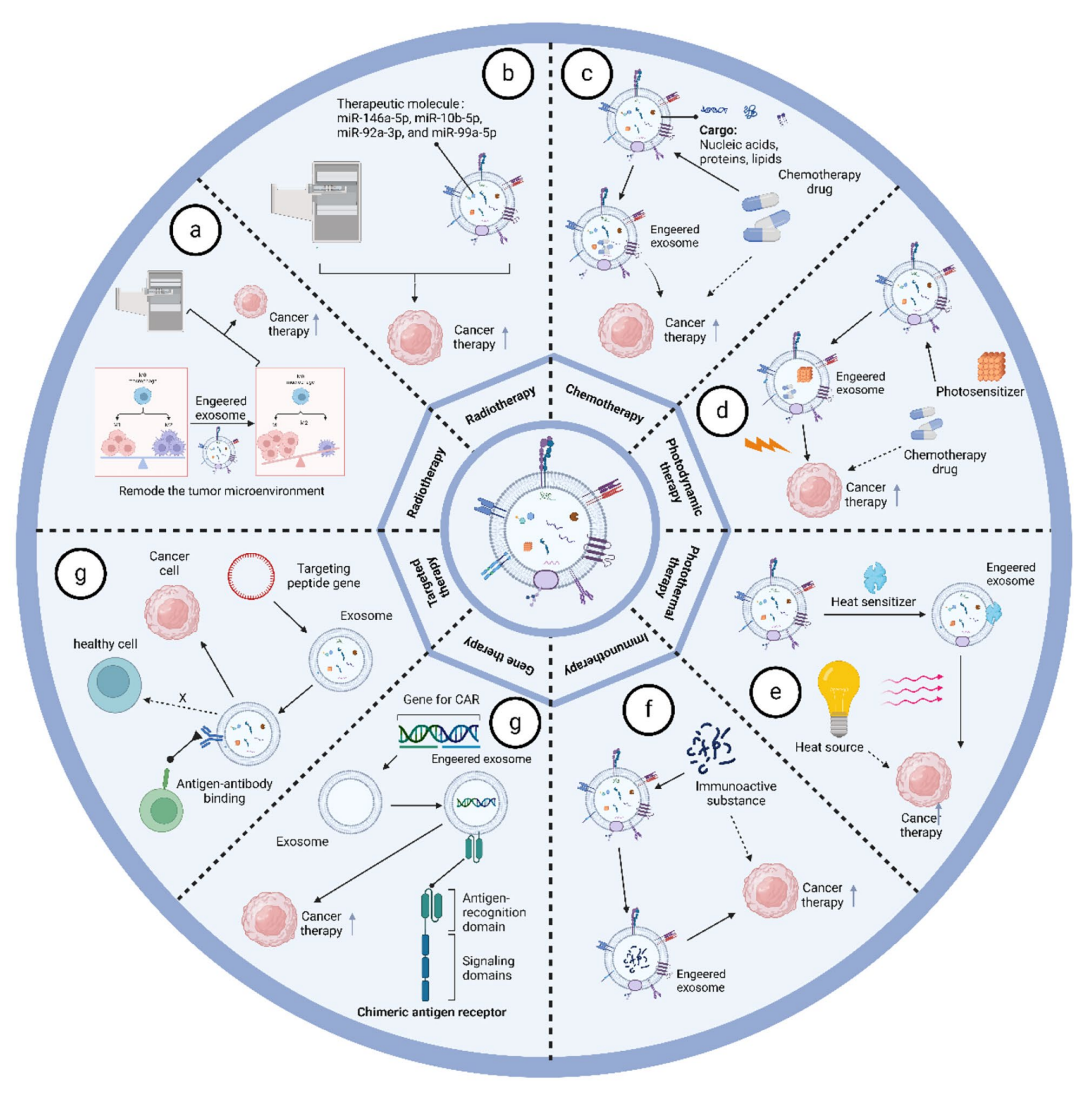
Synergies of engineered natural killer cell-derived exosomes with other tumor therapies. **(a, b)** Combined potentiation of natural killer cell-derived engineered exosomes with radiotherapy. **(c)** Natural killer cell-derived engineered exosomes facilitate chemotherapeutic drug delivery. **(d)** Natural killer cell-derived engineered exosomes are used in combination with photodynamic therapy to promote precise drug release. **(e)** Natural killer cell-derived engineered exosomes are used in combination with photothermal therapy treatment to control the release of tumor drugs. **(f)** Natural killer cell-derived engineered exosomes deliver immune substances into tumor cells. **(g)** Natural killer cell-derived engineered exosomes enter tumor cells and regulate gene expression. **(h)** Natural killer cell-derived engineered exosomes for precision therapy

Radiation therapy plays an important role in the treatment of almost all solid cancers, with wide clinical application and satisfactory results, but there are still some obstacles limiting the efficacy of treatment, such as chemotherapy resistance. An example of the combination of radiation therapy with exosomes of natural killer cell origin is that they contain molecules that activate radio sensitization pathways [65]. Zhu et al. showed that exosomes of natural killer cell origin can enter tumor cells and improve the efficacy of antitumor radiation therapy, such as in melanoma [18]. Combined with microRNA sequencing results of natural killer cell-derived exosomes, we found that natural killer cell-derived exosomes contain molecules such as miR-146a-5p, miR-10b-5p, miR-92a-3p, and miR-99a-5p, which are capable of specifically attenuating tumor radio resistance involving multiple molecular pathways, such as the ATM/ ATR serine/threonine kinases, protein kinase B/mTOR and Janus kinase. Thus, natural killer cell-derived exosomes are able to increase the sensitivity of a wide range of tumors to radiotherapy by transducing miRNAs into tumor cells [66–70] (Fig. 4).

### Chemotherapy

Chemotherapy is a common form of tumor treatment and is often administered systemically due to the lack of targeting of chemotherapeutic agents. Systemic administration of chemotherapeutic agents leads to various TRAEs, including cardiotoxicity and frontal peripheral sensory neuropathy, and reduced anti-tumor efficiency [71–74]. Therefore, combination with engineered exosomes of natural killer cell origin would be a solution to these challenges. Cisplatin is a broad-spectrum chemotherapeutic agent that can be encapsulated into natural killer cell-derived exosomes. Zhao et al. showed that exosomes derived from natural killer cells express protein markers typical of natural killer cells, can be preferentially taken up by SKOV3 cells, and show cytotoxicity to OC cells. Meanwhile, the combined use of the chemotherapeutic drug cisplatin can sensitize drug-resistant ovarian cancer cells to the frontal proliferative effect of cisplatin and achieve significant anti-tumor effects with less damage to major organs [75]. Paclitaxel is another commonly used chemotherapeutic agent, and the results of Gao et al. demonstrated that the use of natural killer cell-derived exosomes in combination with paclitaxel effectively inhibited tumor cell proliferation induced apoptosis and reduced damage to normal cells [76]. In conclusion, combination therapy is able to significantly enhance its tumor targeting and delivery capabilities compared to chemotherapeutic agents alone. At the same time, it can trigger stronger anti-tumor activity and less systemic toxicity (Fig. 4).

### Photodynamic therapy

Photodynamic therapy is a minimally invasive cancer treatment that uses ROS produced by photosensitizers to treat tumors [77]. A distinguishing feature of photodynamic therapy is its ability to sensitize cancer immunotherapy with the controlled release of therapeutic molecules encapsulated in exosomes [78]. An example of photodynamic therapy based on natural killer cell-derived exosomes is Exo^Car/T7^@Micelle designed by Liu et al. Exo^Car/T7^@Micelle is a bionic nano-bomb that facilitates ROS-triggered drug release with the help of the photosensitizer chlorine e6 (Ce6) and ROS-sensitively linked chemical thioketone of bone (TK) to achieve precision strikes against breast cancer tumors through double targeting by to achieve a precise strike against breast cancer tumor cells [16]. Loading photosensitizers into engineered exosomes is the key to achieving precision therapy, and photodynamic therapy, which releases therapeutic molecules from exosomes at a specific time and space compared to conventional administration, is the key to alleviating TRAE. At the same time, we can also use photodynamic therapy to selectively kill tumor cells. Another example is the LASNEO system developed by Huang et al. The LASNEO system is prepared by loading hydrophilic siRNA into natural killer cell-derived exosomes, which are then incubated with a hydrophobic photosensitizer of chlorine e6. Under 660 nm laser irradiation, an effective PDT effect is produced. In addition, ROS disrupts the endolysosomal membrane and promotes the release of siRNA from exosomes, which in turn silences the PD-L1 gene [79]. In conclusion, photodynamic therapy used in combination with exosomes can enhance its anti-tumor effects (Fig. 4).

### Photothermal therapy

Nanomaterial-based photothermal therapies (PTT) have entered clinical studies for some solid tumor types compared to traditional tumor treatments [80–82]. Photothermal therapy does not rely on oxygen and eliminates solid tumors by overcoming the hypoxic tumor microenvironment [83]. A range of photothermal agents have been developed such as carbon nanotubes, and double-based nanoparticles [84, 85]. However, these materials have significant drawbacks, including complex synthesis processes and low biodegradability. Meanwhile, the tumor-killing efficacy of photothermal therapy requires high temperatures in order to ablate tumor tissues and overcome heat shock protein-induced thermotolerance, which is highly susceptible to thermal damage to normal organs near tumors [86]. To address these problems, Liu et al. designed an integrated photothermal therapy based on natural killer cells. A novel PTA was constructed through the coordination of tetrahydroxyanthraquinone and Mn, and by further adsorption of polyetherimide/DNA^zymes^ on the surface, DNA^zymes^@Mn-CONASHs exhibited excellent photothermal conversion, enhanced tumor microenvironmental T1-MRI-directed ability, and thermal durability resistance. At the same time, the researchers used artificially engineered NK cells with HCC-specific targeting of TLS11a-adaptor modifications to obtain engineered exosomes for specific elimination of any possible residual tumor cells after PTT [87]. This combination therapy has achieved excellent and significantly improved anti-tumor efficiency in vivo. Another example of the use of photothermal therapy in conjunction with natural killer cell-derived exosomes is the study by Li et al. Granzyme B is a serine protease enriched in natural killer cells and their exosomes. Granzyme B is able to enter target cells in the presence of perforin and induce apoptosis in target cells [88–91]. Li et al. designed exosome-based and photothermal-sensitive liposome fusion nanoplatforms with excellent cascade tumor-targeting and cytotoxicity properties under laser irradiation, showing excellent therapeutic efficacy [92]. In conclusion, the combination of exosomes and photothermal therapy significantly increases the penetration capacity of photothermal therapy and reduces the use of laser dose compared with conventional photothermal therapy, thus enhancing the tumor treatment outcome (Fig. 4).

### Immunotherapy

Immunotherapy refers to the method of causing the body to produce an immune response through active or passive means, specifically and efficiently exerting the function of suppressing and killing tumor cells. Unlike traditional treatment methods, immunotherapy does not kill the cells directly, but mobilizes the immune cells in the interior of the body. Given the properties of natural killer cell-derived exosomes, the combined use of engineered exosomes with immunotherapy is of increasing interest. For example, Shoae et al. showed that natural killer cell-derived exosomes were able to modify the expression of the natural cytotoxicity receptor of NK cells in vivo, making them more toxic to neuroblastoma cells, which led to much more effective immunotherapy [93]. Natural killer cell-derived exosomes also stimulate T cells, monocytes, and substances that act on the TGF-β pathway, thereby attenuating immunosuppression and enhancing immunotherapy [94–96]. Natural killer cell-derived exosomes can carry specific targeting molecules into tumor cells to perform their killing function, in addition to their own tumor-killing function. Neviani et al. showed that exosomes released by NK cells contain miR-186, a tumor growth inhibitor. miR-186 interferes with TGF-β-mediated inhibition of NK function by decreasing NB cell migration and proliferation through the targeting and down-regulation of TGFβR1, TGFβR2, and SMAD3. In addition, Neviani et al. blocked TGF-β-mediated inhibition by specifically delivering miR-186 mimics to NK cells using nanoparticles loaded with CD56 antibody. From these results, it is clear that natural killer cell-derived exosomes maintain their properties even under immunosuppressive conditions, suggesting that they can be used in combination with immunotherapy to improve the prognosis of patients with high-risk neuroblastoma [64]. The tumor immunosuppressive microenvironment hampers the effectiveness of immunotherapy, and the use of natural killer cell-derived exosomes to remodel the tumor immune microenvironment has received increasing attention. The tumor immunosuppressive microenvironment hinders the effectiveness of immunotherapy, and the use of natural killer cell-derived exosomes to remodel the tumor immune microenvironment has received increasing attention.Huang et al. developed a light-activated silencing NK-derived exocytosis (LASNEO) system. Not only achieved effective PDT, but also promoted macrophage reprogramming to M1 phenotype and mature dendritic cells, and activated CD4 + T cells and CD8 + T cells in TME through PD-L1 inhibition [79]. In summary, our understanding of the tumor microenvironment has advanced considerably over the past few decades, which provides a wealth of potential targets for engineering exosomes. In the future, the use of natural killer cell-derived exosomes in combination with immunotherapy will be a great addition to the field of cancer treatment efficacy (Fig. 4).

### Gene therapy

Gene therapy is a biological treatment method in which exogenous normal genes are introduced into target cells through gene transfer technology to correct or compensate for diseases caused by genetic defects and abnormalities, and ultimately achieve therapeutic goals [97, 98]. Exosomal non-coding RNAs (e.g. miRNAs) have shown great therapeutic potential in a variety of cancers and can be used as gene expression vectors for tumor therapy [99–101]. However, the poor controllability of the expression level and location of expression is a major obstacle to the use of non-coding RNAs for gene therapy at present. For example, Wang et al. delivered miR-1249-3p using natural killer cell-derived exosomes to regulate insulin resistance by directly targeting SKOR1 to regulate the formation of the ternary complex SMAD6/MYD88/SMURF1 and inhibit the TLR4/NF-κB signaling pathway to mediate glucose homeostasis [61]. In another study, Hu et al. delivered miR-207 using natural killer cells. miR-207 interacted with TLR4 and inhibited NF-κB signaling in astrocytes, reducing the release of pro-inflammatory cytokines and alleviating depressive symptoms [102]. Thus, the therapeutic efficacy of non-coding RNAs in disease has been demonstrated, and the combination of gene therapy with exosomes of natural killer cell origin has improved therapeutic efficacy and accelerated the use of gene therapy in clinical trials (Fig. 4).

### Targeted therapy

The targeted therapy of cancer refers to the design of drugs at the cellular or molecular level against known cancer-causing sites, which specifically enter the receptor cells and act to cause the specific death of tumor cells without affecting the normal cells surrounding the tumor. In recent years, CAR-T cell-derived exosomes have gained a great deal of attention due to their more potent anti-tumor effects [103, 104]. Recently, the study by Liu et al. successfully applied chimeric antigen receptors to natural killer cell-derived exosomes, aiming to enhance the effectiveness of antitumor therapy by disrupting the iron death defense mechanism. The modification of transferrin receptor-binding peptide and the expression of CAR on the exosome surface successfully crossed the blood-brain barrier and facilitated the release of therapeutic molecules at specific sites and times [16]. Compared to CAR-T cell-derived exosomes, CAR-NK cell-derived exosomes offer several advantages: safer, multiple tumor-killing mechanisms, more widely available, and relatively homogeneous and efficient quality [105] (Fig. 4).

## Conclusion

Currently, many studies on exosomes of natural killer cell origin have demonstrated their potential as drug delivery, as well as their ability to be used in combination with other tumor therapeutics and to improve therapeutic efficacy. However, exosomes of natural killer cell origin also have certain limitations, such as the differentiation potential of natural killer cells, and the exosomes secreted by them may be heterogeneous, affecting the therapeutic effect of the disease. The emergence of exosome engineering technology provides new methods and ideas to solve this problem, and how to choose the appropriate exosome modification strategy will be a new direction for future tumor therapy. In conclusion, in view of the current maturity of engineered exosome technology, natural killer cell-derived exosomes containing engineered modifications will certainly provide a new method and idea for tumor therapy.

## Data Availability

No datasets were generated or analysed during the current study.

## Abbreviations

NK: Natural killer
CLP: lymphoid progenitors
CILCP: ILC precursor stage
NKP: NK progenitors
PDT: Photodynamic therapy
Ce6: Chlorine e6
GVHD: Graft-versus-host disease
ROS: Reactive oxygen species
CAR: Chimeric Antigen Receptor
PTT: Photothermal therapies
